# An *in vitro* pressure model towards studying the response of primary retinal ganglion cells to elevated hydrostatic pressures

**DOI:** 10.1038/s41598-019-45510-7

**Published:** 2019-06-21

**Authors:** Jing Wu, Heather Kayew Mak, Yau Kei Chan, Chen Lin, Cihang Kong, Christopher Kai Shun Leung, Ho Cheung Shum

**Affiliations:** 1 Department of Mechanical Engineering, University of Hong Kong, Hong Kong, China; 2 HKU-Shenzhen Institute of Research and Innovation (HKU-SIRI), Shenzhen, Guangdong 518000 China; 3 Department of Ophthalmology and Visual Sciences, The Chinese University of Hong Kong, Hong Kong Eye Hospital 147K Argyle Street Kowloon, Hong Kong, China; 4 Department of Electrical and Electronic Engineering, University of Hong Kong, Hong Kong, China

**Keywords:** Lab-on-a-chip, Neurophysiology

## Abstract

Glaucoma is a leading cause of blindness characterized by progressive degeneration of retinal ganglion cells (RGCs). A well-established risk factor for the development and progression of glaucoma is elevation of intraocular pressure (IOP). However, how elevated IOP leads to RGC degeneration remains poorly understood. Here, we fabricate a facile, tunable hydrostatic pressure platform to study the effect of increased hydrostatic pressure on RGC axon and total neurite length, cell body area, dendritic branching, and cell survival. The hydrostatic pressure can be adjusted by varying the height of a liquid reservoir attached to a three-dimensional (3D)-printed adapter. The proposed platform enables long-term monitoring of primary RGCs in response to various pressure levels. Our results showed pressure-dependent changes in the axon length, and the total neurite length. The proportion of RGCs with neurite extensions significantly decreased by an average of 38 ± 2% (mean ± SEM) at pressures 30 mmHg and above (p < 0.05). The axon length and total neurite length decreased at a rate of 1.65 ± 0.18 μm and 4.07 ± 0.34 μm, respectively (p < 0.001), for each mmHg increase in pressure after 72 hours pressure treatment. Dendritic branching increased by 0.20 ± 0.05 intersections/day at pressures below 25 mmHg, and decreased by 0.07 ± 0.01 intersections/day at pressures above 25 mmHg (p < 0.001). There were no significant changes in cell body area under different levels of hydrostatic pressure (p ≥ 0.05). Application of this model will facilitate studies on the biophysical mechanisms that contribute to the pathophysiology of glaucoma and provide a channel for the screening of potential pharmacological agents for neuroprotection.

## Introduction

Glaucoma, a leading cause of blindness worldwide^1–4^, is a neurodegenerative disease of the optic nerve characterized by a progressive degeneration of retinal ganglion cells (RGCs)^4–7^. A well-established risk factor for the development and progression of glaucoma is the elevation of intraocular pressure (IOP)^2,8–14^. It is largely unclear how IOP elevation leads to degeneration of RGCs in glaucoma, although pressure-induced lamina cribrosa deformation, extracellular matrix remodeling, ocular blood flow dysregulation and ischemia, impairment in the delivery of neurotrophic factors, and subsequent mitochondrial dysfunction and metabolic failure in the RGCs have been implicated in the pathogenesis of glaucoma^15–20^.

*In vitro* microfluidic devices have brought significant progress in the field of biomedical research over the past years^21–24^. Poly(dimethylsiloxane) (PDMS) microfluidic chambers are favourable for *in vitro* cell culture studies due to its high biocompatibility. PDMS is oxygen permeable, optically transparent, stable at physiological temperatures, and can be efficiently produced at low costs^25^. As PDMS microfluidic chambers can be produced via imprinting, chamber size and dimensions can be customized to suit specific experimental conditions, allowing precise control over the culture environment. Unlike *in vivo* models, an *in vitro* PDMS microfluidic platform allows acquisition of cellular responses at a single cell level^22,26–30^. The malleability of PDMS microfluidic chambers not only allows micro-scaled volumes, which reduces the amount of culture medium required, but also facilitates the design of various models to simulate biophysical mechanics, such as shear stress, tension, and compression^22,26,29,31,32^. Both cell lines and primary cells have been investigated using the *in vitro* PDMS microfluidic chamber platforms^28,33^. Although the rapid proliferation of cell lines allows an ease of culture maintenance with low cell death rates, their genetic, phenotypic, structural, and functional characteristics often differ from those *in vivo*^28,34,35^. The utilization of primary cells isolated directly from the targeted tissue^28,36^, therefore, provides a more accurate representation of cellular responses to treatment and stimulation^37–40^.

Recent studies have attempted to evaluate the response of RGCs to increased pressure by using centrifugal forces^41^ and hyperbaric pressure^42^ for mechanical stimulation. However, neither condition is physiologically representative of the cellular environment within the eye because they induce high shear stress onto the cultured cells. In this study, we designed an *in vitro* PDMS microfluidic chamber that can provide a platform of the eye with tunable pressure for the investigation of pressure-induced stress on primary RGCs. The cell loading area of the PDMS microfluidic chamber was designed to have a trench shape, which minimizes the amount of shear stress during the loading of cells and replenishment of medium^43–45^. As the daytime IOP of rats has been reported to be between 10 mmHg and 20 mmHg^10,46^, various hydrostatic pressure levels below and above 20 mmHg were applied to primary RGCs isolated from post-natal day 6 Sprague-Dawley (SD) rats: 0 mmHg, 10 mmHg, 20 mmHg, 25 mmHg, 30 mmHg, 40 mmHg, and 50 mmHg. Images were acquired for three consecutive days after pressure elevation using an optical microscope and the axon length, total neurite length, neurite branching complexity, and soma size of RGCs were measured.

## Results and Discussion

### Platform design and operation

**Figure 1:**
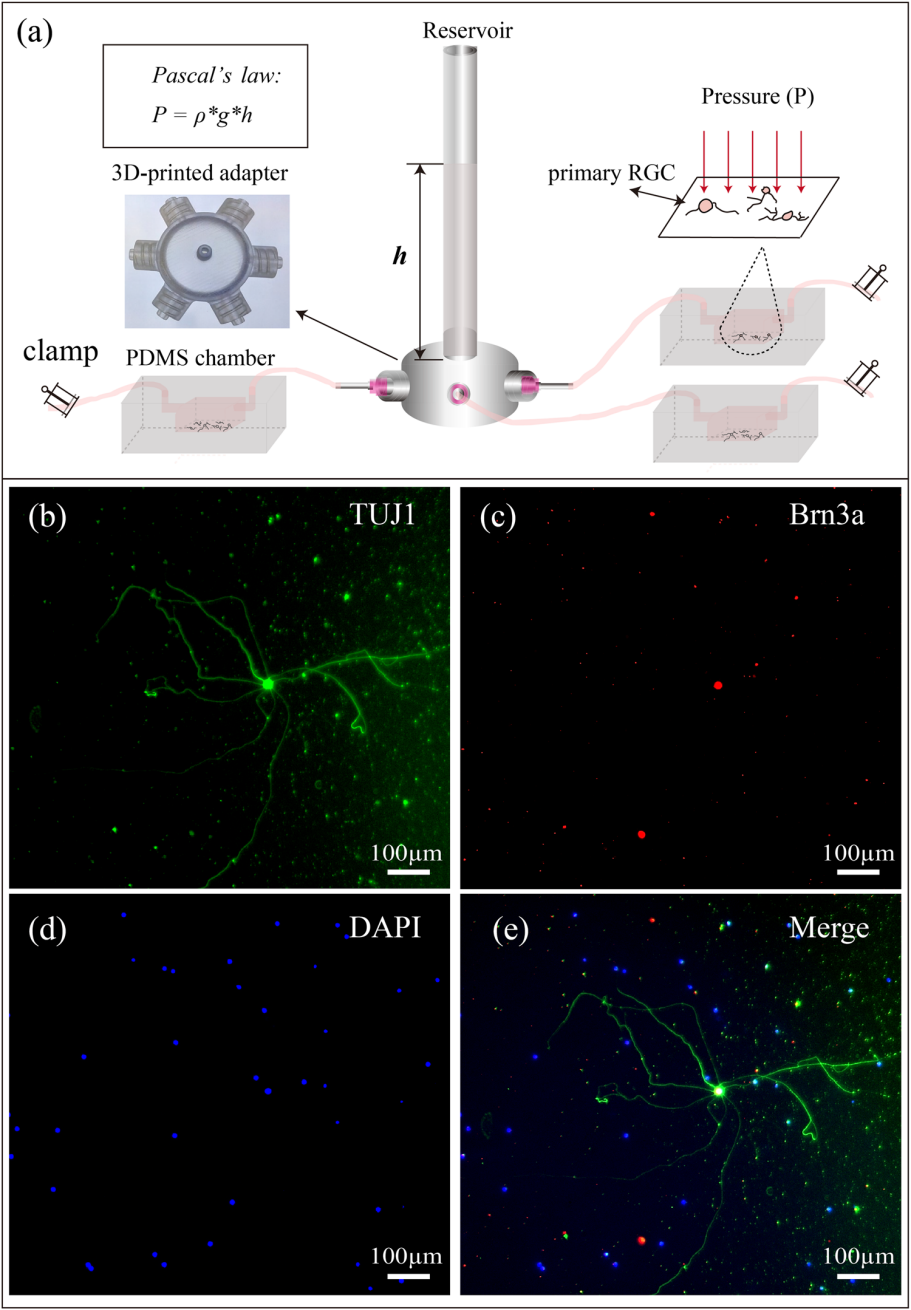
(**a**) A schematic diagram of the devised pressure apparatus for the application of adjustable hydrostatic pressure to primary RGCs using Pascal’s law. Multiple PDMS chambers, containing primary RGC cultures, are connected to a 3D-printed adapter via silicone tubing, and subjected to various hydrostatic pressures depending on the height h of FC-40 within the attached transparent reservoir. (**b**–**e**) Representative fluorescence images of primary RGCs cultured inside a PDMS chamber at day 3 *in vitro*. RGCs were positively stained with TUJ1 (green), BRN3A (Red) and DAPI (blue), which are neuronal-specific, RGC-specific and nucleus markers, respectively.

We designed a 3D-printable adapter that can simultaneously connect up to 6 PDMS devices via silicone tubing (Fig. 1a). The outlets of the 3D-printed adapter are equally spaced in a radial pattern and compatible with commercially available needle tips. The availability of multiple outlets per adapter allows all RGCs connected to the same adapter to be subjected to the same pressure applied throughout the experiment. Hydrostatic pressure is adjusted by varying the height *h* of Fluorinert^TM^ FC-40 (Sigma-Aldrich, Saint Louis, MO, USA) in the reservoir. Fluorinert^TM^ FC-40 has been demonstrated to be highly biocompatible^47^, with a low solubility in biological reagents^48^ due to its high density (1.85 * 10^3^ kg/m^3^). The applied hydrostatic pressure is calculated according to Pascal’s law, ${\rm{\Delta }}P={\rm{\rho }}\ast {\rm{g}}\ast ({\rm{\Delta }}h)$ where ΔP is the hydrostatic pressure (Pa), ρ is the density of FC-40 (1.85 * 10^3^ kg/m^3^) and Δh is the difference in height *h* of FC-40 relative to the surface of the culture medium (m). The applied hydrostatic pressures were 0 mmHg, 10 mmHg, 20 mmHg, 25 mmHg, 30 mmHg, 40 mmHg, and 50 mmHg (referred as defined pressures below), which corresponded to FC-40 liquid heights of 0 cm, 7.4 cm, 14.9 cm, 18.6 cm, 22.3 cm, 29.7 cm, and 37.2 cm, respectively. To validate the applied pressures, the pressures inside the PDMS devices were also measured using OPP-M250 Packaged Pressure Sensor (Opsens Inc. Canada), and the measured pressures were comparable to applied pressures: 0.4 ± 0.3 mmHg, 9.8 ± 0.2 mmHg, 20.2 ± 0.6 mmHg, 25.9 ± 0.6 mmHg, 29.7 ± 1.1 mmHg, 38.8 ± 0.6 mmHg, and 48.2 ± 0.9 mmHg, respectively (p ≥ 0.05)). The percentage difference between the theoretical and measured values from 10, 20, 25, 30, 40 and 50 mmHg are −2.0 ± 1.2%, 0.8 ± 1.8%, −0.3 ± 1.3%, −1.0 ± 2.1%, −3.1 ± 0.8%, and −3.5 ± 1.0% (±SEM) respectively. Possible sources of error include precision of the sensor, temperature difference, density difference, as well as manual measurement error. All theoretical and experimental pressure readings are shown in Fig. S1.

**Figure 2:**
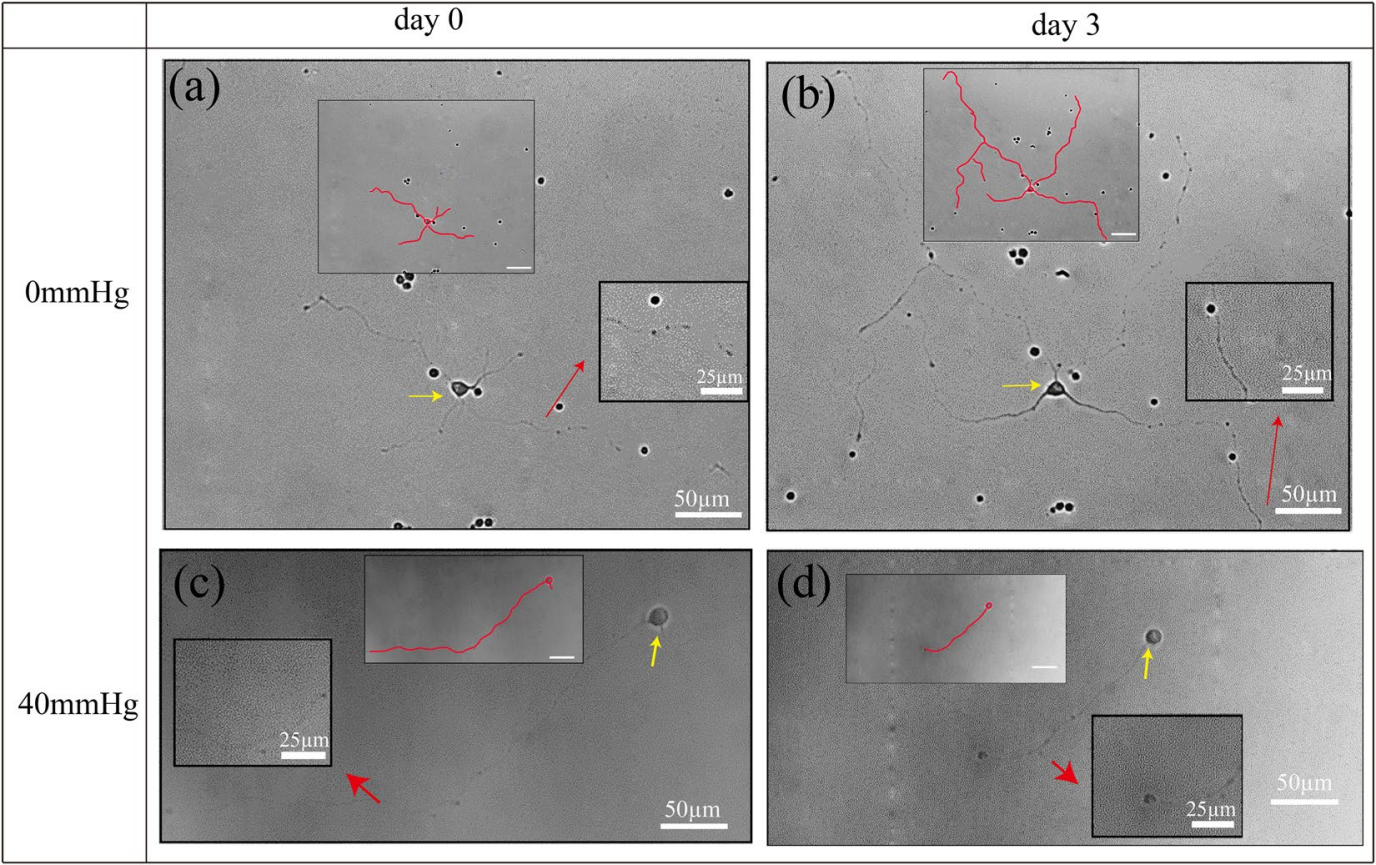
Representative phase contrast images and manually traced cell with neurites showing the same primary RGCs before and after 3 days of pressure elevation. (**a**) and (**c**) are images before pressure elevation, (**b**) and (**d**) are images after 3 days of 0 mmHg and 40 mmHg pressure elevation, respectively. Yellow and red arrows indicate the cell body and neurites, respectively.

At 3 days *in vitro* without the applied pressure, the RGCs in PDMS chambers display healthy neuronal morphology with a large, circular cell body and long extended neurites (RGC isolation and culture methodology are illustrated in Supporting Information). Fluorescent immunostaining validated the RGC identity of the cultured cell population by showing positive expressions for both tubulin ß-III (TUJ1), a neuron-specific marker, and brain-specific homeobox domain protein 3 A (BRN3A), an RGC-specific transcription factor located in the cell nucleus (Fig. 1b–d) (Immunocytochemistry method is provided in Supporting Information). To study the response of primary RGCs to different pressure levels, PDMS chambers were connected to liquid reservoirs with different heights of FC-40, as illustrated in Fig. 1a. The entire pressure apparatus was placed inside an incubator maintained at 37 °C with 5% carbon dioxide (CO_2_). RGCs were subjected to defined pressures, for three consecutive days. Phase contrast images were acquired before and after pressure treatment using an inverted optical microscope (Fig. S2). RGC neurite lengths increased (Fig. 2a) and decreased (Fig. 2b) under pressure levels of 0 mmHg and 40 mmHg, respectively. The pressure-dependent change in neurite lengths suggested that the designed apparatus could simulate an environment that can be used to study the integrity of RGCs in different levels of pressure elevation^19,49^.

**Figure 3:**
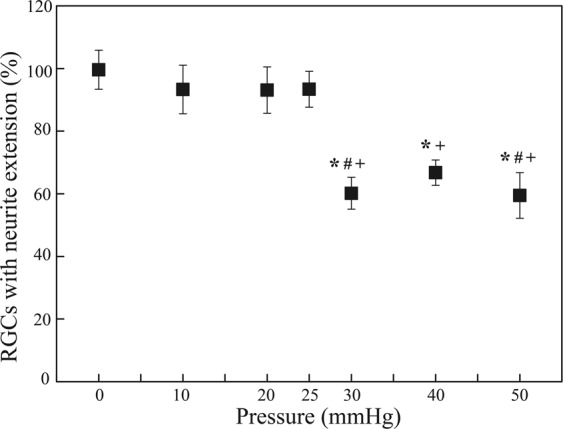
The proportion of RGCs with neurite extensions (%) 3 days after applying a hydrostatic pressure of 0 mmHg (n = 22 devices, n_0,RGCs_ = 993), 10 mmHg (n = 4 devices, n_0,RGCs_ = 106), 20 mmHg (n = 6 devices, n_0,RGCs_ = 534), 25 mmHg (n = 7 devices, n_0,RGCs_ = 503), 30 mmHg (n = 11 devices, n_0,RGCs_ = 552), 40 mmHg (n = 9 devices, n_0,RGCs_ = 370), and 50 mmHg (n = 11 devices, n_0,RGCs_ = 432 RGCs) for 72 hours. Data are in mean ± SEM. *, # and + indicate statistically different results from 0 mmHg (P < 0.05), 10 mmHg (P < 0.05), and 20 mmHg (P < 0.05) respectively by one-way ANOVA followed by Bonferroni post-hoc test. N_0,RGCs_ represents the number of RGCs with neurite extensions at the baseline.

After 3 consecutive days of a hydrostatic pressure of 25 mmHg or below, the total number of primary RGCs with neurite extensions remained the same (p ≥ 0.05). At pressures above 25 mmHg, however, the proportions of RGCs with neurite extensions decreased significantly compared with the baseline (p < 0.05). They were 60.2 ± 5.1%, 66.8 ± 4.0%, and 59.5 ± 7.3% (mean ± SEM) at 30 mmHg, 40 mmHg, and 50 mmHg respectively. By contrast, they were 99.6 ± 6.2%, 93.3 ± 7.7%, 93.1 ± 7.4%, and 93.3 ± 5.7% at 0 mmHg, 10 mmHg, 20 mmHg, and 25 mmHg, respectively (Fig. 3).

**Figure 4:**
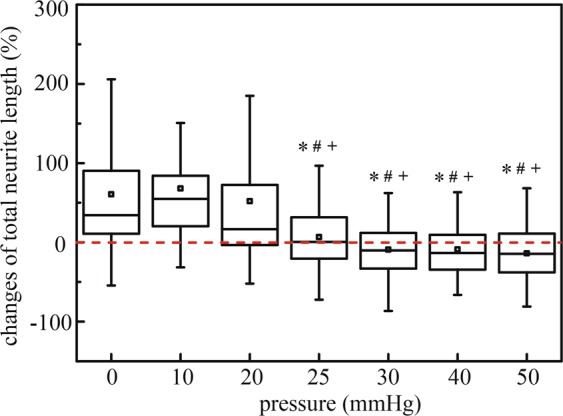
Box plot of percentage of total neurite length changes after applied hydrostatic pressures of 0 mmHg (n = 155 RGCs), 10 mmHg (n = 67 RGCs), 20 mmHg (n = 109 RGCs), 25 mmHg (n = 77 RGCs), 30 mmHg (n = 76 RGCs), 40 mmHg (n = 80 RGCs), and 50 mmHg (n = 74 RGCs) for 72 hours. Box plot whiskers indicate the maximum and minimum values of the data. The symbols *, # and + indicate statistically different results from 0 mmHg (P < 0.05), 10 mmHg (P < 0.05), and 20 mmHg (P < 0.05) respectively by one-way ANOVA followed by Bonferroni post-hoc test.

All RGC neurites were then manually traced and measured by a custom computer program written in MATLAB (Fig. S2) (Cell image analysis and quantitative analysis method are depicted in Supporting Information). Figure 4 shows the percentage change in total neurite length (i.e. the difference in total neurite length on day 3 minus the length on day 0, divided by the length measured on day 0) at different pressure levels. At pressures of 0 mmHg, 10 mmHg, and 20 mmHg, total neurite lengths increased by 118.96 ± 17.46 μm, 78.91 ± 15.88 μm, and 45.27 ± 10.24 μm (mean ± SEM), respectively, after 72 hours of pressure treatment. However, under pressures of 25 mmHg, 30 mmHg, 40 mmHg, and 50 mmHg, total neurite lengths significantly decreased after 72 hours of pressure elevation by 7.21 ± 7.79 μm, 26.00 ± 9.97 μm, 52.76 ± 13.58 μm and 64.05 ± 15.87 μm, respectively. Overall, the total neurite length decreased 4.07 ± 0.34 μm for each mmHg increase in pressure, while controlling for the differences in baseline total neurite lengths between pressure groups (linear mixed model, p < 0.001). Our data demonstrate that RGCs begin to degenerate at hydrostatic pressures 25 mmHg or above, with no signs of RGC degeneration at hydrostatic pressures 20 mmHg or below. This suggests the existence of a critical threshold of pressure beyond which RGC would degenerate.

**Figure 5:**
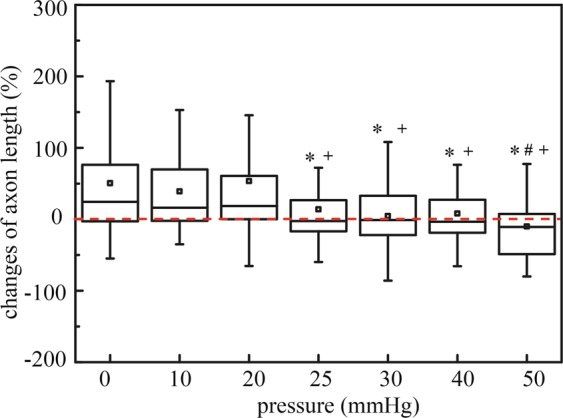
Box plot of axon length after applied hydrostatic pressures of 0 mmHg (n = 155 RGCs), 10 mmHg (n = 67 RGCs), 20 mmHg (n = 109 RGCs), 25 mmHg (n = 77 RGCs), 30 mmHg (n = 76 RGCs), 40 mmHg (n = 80 RGCs), and 50 mmHg (n = 74 RGCs) for 72 hours. Box plot whiskers indicate the maximum and minimum values of the data. The symbols *, # and + indicate statistically different results from 0 mmHg (P < 0.05), 10 mmHg (P < 0.05), and 20 mmHg (P < 0.05) respectively by one-way ANOVA followed by Bonferroni post-hoc test.

The axons of RGCs, which are the sole retinal neuron projections that form the optic nerve connecting the eye to the brain^50^, was defined as the longest neurite extending from the RGC cell body. Figure 5 shows the percentage change in axon length (i.e. the difference in axon length on day 3 minus the length on day 0, divided by the length measured on day 0) at different pressure levels. Similar to the change in total neurite length measurements, under pressures of 0 mmHg, 10 mmHg, and 20 mmHg, axon lengths increased by 52.29 ± 7.75 μm, 24.12 ± 8.67 μm, and 19.01 ± 3.82 μm, respectively (mean ± SEM), after 72 hours of pressure treatment. Under 25 mmHg and 30 mmHg, axon length only increased by 3.26 ± 4.15 μm, and 3.46 ± 7.70 μm, respectively; and under 40 mmHg and 50 mmHg, axon length significantly decreased by 18.57 ± 10.32 μm and 30.67 ± 9.95 μm, respectively. Overall, the axon length decreased by 1.65 ± 0.18μm for each mmHg increase in pressure, while controlling for the differences of baseline axon lengths between pressure groups (linear mixed model, p < 0.001).

**Figure 6:**
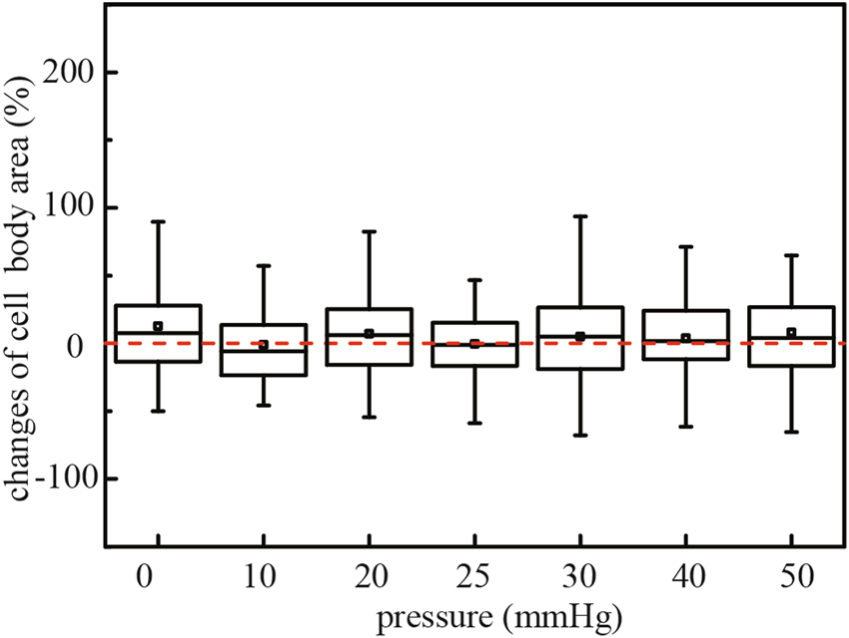
Box plot of cell body area changes after applied hydrostatic pressures of 0 mmHg (n = 155 RGCs), 10 mmHg (n = 67 RGCs), 20 mmHg (n = 109 RGCs), 25 mmHg (n = 77 RGCs), 30 mmHg (n = 76 RGCs), 40 mmHg (n = 80 RGCs), and 50 mmHg (n = 74 RGCs) for 72 hours. Box plot whiskers indicate the maximum and minimum values of the data. The symbols *, # and + indicate statistically different results from 0 mmHg (P < 0.05), 10 mmHg (P < 0.05), and 20mmmHg (P < 0.05) respectively by one-way ANOVA followed by Bonferroni post-hoc test.

The change of RGC cell body area was also presented as percentage changes calculating by dividing the difference in cell body area on day 3 and on day 0 by the area measured on day 0 (Fig. 6). There were no observable pressure induced changes in RGC cell body area under all tested pressure levels (p ≥ 0.05). The changes of cell body area under defined pressures were 12.63 ± 2.95%, -1.1 ± 3.01%, 6.99 ± 2.99%, -0.53 ± 2.88%, 4.83 ± 4.27%, 3.80 ± 3.63%, and 7.97 ± 4.33%, respectively (mean ± SEM) (Fig. 6).

**Figure 7:**
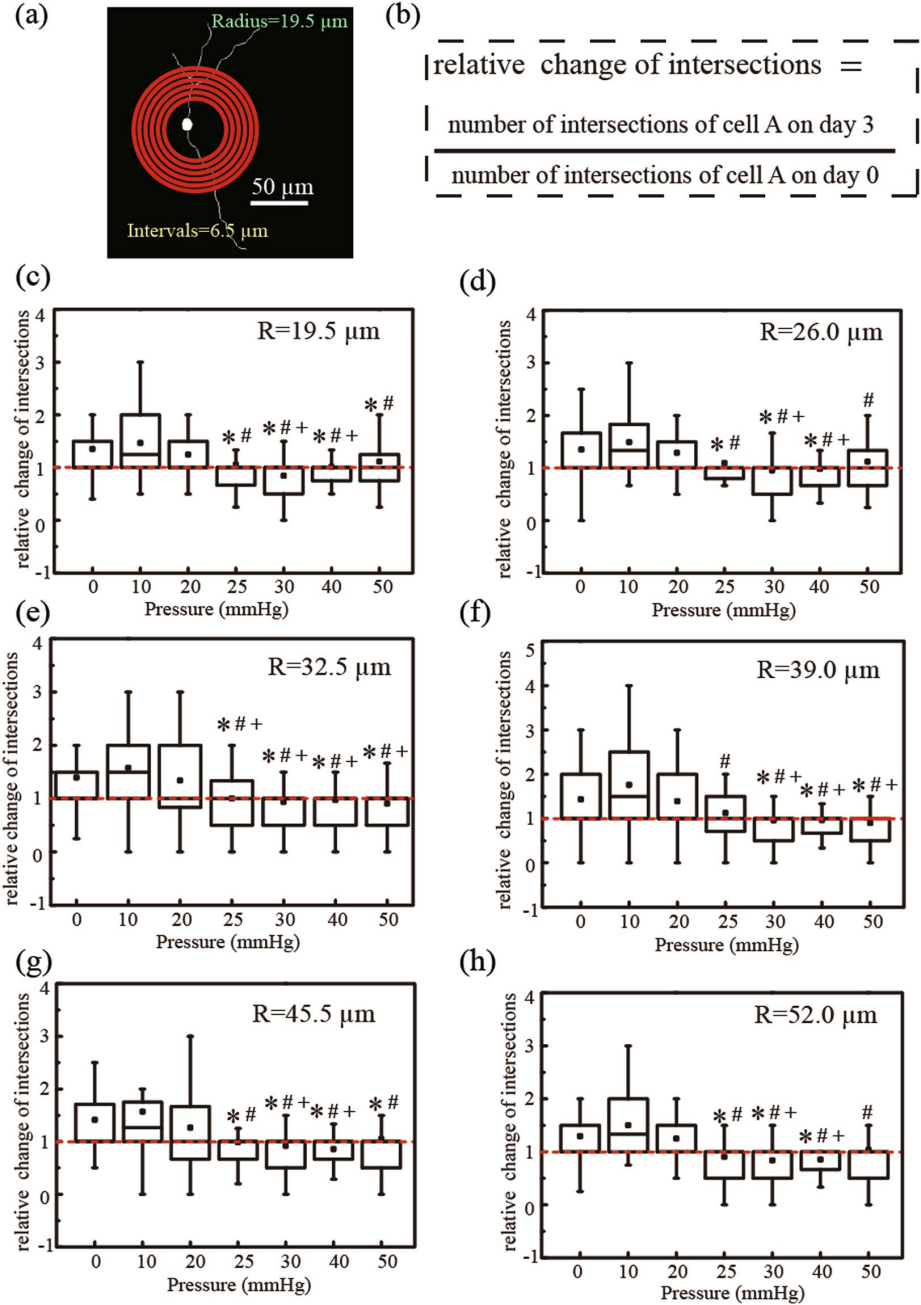
(**a**) Sholl analysis of primary RGCs, (**c**–**h**) Box chart of the relative number of intersections between neurites and concentric circles(R = 19.5 µm, 26.0 µm, 32.5 µm, 39.0 µm, 45.5 µm and 52.0 µm)after applied hydrostatic pressures of 0 mmHg (n = 155 RGCs), 10 mmHg (n = 67 RGCs), 20 mmHg (n = 109 RGCs), 25 mmHg (n = 77 RGCs), 30 mmHg (n = 76 RGCs), 40 mmHg (n = 80 RGCs), and 50 mmHg (n = 74 RGCs) on day 3. Box plot whiskers indicate the maximum and minimum values of the data. The symbols *, # and + indicate statistical significance relative to 0 mmHg (P < 0.05), 10 mmHg (P < 0.05), and 20 mmHg (P < 0.05), respectively, using One-way ANOVA followed by Bonferroni Post-hoc Test.

Using a Sholl analysis, RGC neurite branching complexity can be measured by quantifying the total number of intersections between the neurites of an individual RGC and a series of concentric circles at fixed distances away from the cell soma^50,51^. The relative change in RGC branching complexity was calculated by normalising the number of intersections on day 3 to day 0 (see equation in Fig. 7b). While controlling for repeated measurements from the same cell, linear mixed modelling showed that at pressures below 25 mmHg, the total number of intersections per cell increased over time (0.20 ± 0.05 intersections/day, p ≤ 0.001); while at pressures above 25 mmHg, the total number of intersections per cell decreased over time (-0.07 ± 0.01 intersections/day, p ≤ 0.026). Although the total number of intersections per cell showed a slight decrease (-0.01 ± 0.02 intersections/day) at 25 mmHg, it was not yet significant (p = 0.776). The increase in neurite branching at pressures < 25 mmHg suggests that dendritic complexity increases at normal ranges of physiological pressures, and the significant decrease at pressures > 25 mmHg indicates that dendritic branching complexity decreases at pressures above physiologically normal ranges.

In this study, we designed an *in vitro* hydrostatic pressure apparatus to study the morphology of primary RGCs in response to physiologically abnormal pressures. Notably, our finding is different from a previous study demonstrating no RGC degeneration in response to pressure elevation using a hyperbaric chamber^42^. Our microfluidic chamber model can provide an efficient framework to study the relationship between intraocular pressure and RGC degeneration. That the pressure levels were associated with the total neurite length and axon length of RGCs corroborates findings from clinical studies in glaucoma patients demonstrating that the rate of retinal nerve fiber layer thinning increases with IOP^52^.

Our data indicate the devised pressure apparatus can become a tunable pressure platform for the investigation of short-term and long-term responses of primary RGCs to pressure elevation and fluctuation. We are developing a pressure control system that can apply fluctuations of pressure to RGCs over time. Moreover, the PDMS surface on which the primary RGCs were plated has the relative elastic properties within range of the surrounding ocular environment of RGCs in the eye, with a Young’s modulus similar to that of Bruch’s membrane, the layer at which RGCs are situated above, and smaller than that of the ocular scleral strip^53–55^. IOP fluctuates depending on circadian rhythm and pulsatile blood pressure, making it difficult to simulate IOP fluctuation in an experimental model to investigate its effect on RGCs. Our designed pressure apparatus would have the flexibility of inducing a wide range of hydrostatic pressures with precision and control. The current knowledge on neurodegeneration of the retina due to abnormal intraocular pressure is still limited^5,56,57^, albeit the growing understanding on the inner limiting membrane, formed by astrocytes and Müller cells, playing a role in retinal degeneration, in which its addition would make the PDMS setup more physiologically relevant. However, despite the complicated nature of the neurological system, key features associated with retinal degeneration were still able to be observed using our simple system with primary RGCs alone.

Although it has been demonstrated that 70 mmHg of pressure outside the cell membrane of K562 cells, a myelogenous leukemia cell line, quickly passes to the intracellular fluidic environment with little to no change in membrane tension^58^, the cell membrane permeability of RGCs to elevated pressures remains unknown and more studies will be needed to further elucidate this phenomenon under lower pressure ranges that are more clinically relevant (e.g. 10 to 30 mmHg). However, the response of RGCs to changes in hydrostatic pressure has been widely documented. There are studies that demonstrate pressure-induced activation of ion channels in the cell membrane^58,59^, and elevated pressure has been shown to activate transient receptor potential banilloid 1(TRPV1) in primary RGCs, which is related to the release of Ca^2+^ from intracellular stores^60,61^. Other cellular responses of RGCs to increased pressures include mitochondrial dysfunction^16–18^, extracellular matrix remodeling^19^, and biomolecular changes (e.g. the decline of NAD + and glutathione)^20^. An advantageous design of the PDMS chamber is its optical transparency that allows live cell fluorescent imaging studies. This will facilitate live cell tracking of biomolecular changes in RGCs upon pressure elevation, introducing a wider range of experiments that can be performed to study the biophysical mechanisms that contribute to the pathophysiology of glaucoma.

Unlike previous studies which examined the impact of hydrostatic pressure on transformed cell lines^62^, our study has established an *in vitro* platform to investigate the impact of hydrostatic pressure on primary RGCs. Remarkably, whereas the axon and neurites of the RGCs extended at pressure levels below 25 mmHg, they decreased at a rate of 1.65 ± 0.18 µm and 4.07 ± 0.34 μm, respectively, for each mmHg increase in pressure above 25 mmHg. This finding recapitulates a key characteristic of human glaucoma in which the risk of optic nerve degeneration increases with the levels of IOP. We did not obtain oxygen tension measurements and it is unclear whether or to what degree hypoxia was connected to RGC degeneration in our system. Nevertheless, the proposed platform shows unique advantages by isolating pressure from other parameters that can be associated with RGC degeneration in glaucoma, such as cell-cell communication, extracellular matrix and glial cell involvement^21,24,41,57^. This tunable hydrostatic pressure system would be useful to investigate the cellular and molecular pathways of RGC degeneration in response to pressure elevation.

## Conclusions

In summary, we devised an *in vitro* platform with adjustable hydrostatic pressure levels that simulates a range of physiologically relevant intraocular pressure levels. This platform allows (1) optical transparency for clear visualisation of primary RGCs at a single-cell level, (2) long-term monitoring and quantification of the morphological changes of primary RGCs over time, and (3) facile changes of applied pressure by varying the height of the liquid column. We show that there is a critical pressure threshold at approximately 25 mmHg, beyond which RGCs become vulnerable to cell death. Specifically, at pressure levels below 25 mmHg, RGCs continue to extend neurite length and increase in branching complexity over time. By contrast, at pressure levels at or above 25 mmHg, RGCs no longer extend neurites, but begin to degenerate and decrease in neurite length and branching complexity. This study demonstrates the applicability of this platform to simulate the biophysical relationship between elevated intraocular pressure and the degeneration of retinal ganglion cells. Establishment of this device will greatly facilitate further research on the intracellular mechanisms of RGCs in response to the dynamic fluid mechanics of the eye, which have yet to be investigated.

## Experimental Methods

### Fabrication of trench-shaped PDMS device by imprinting

**Figure 8:**
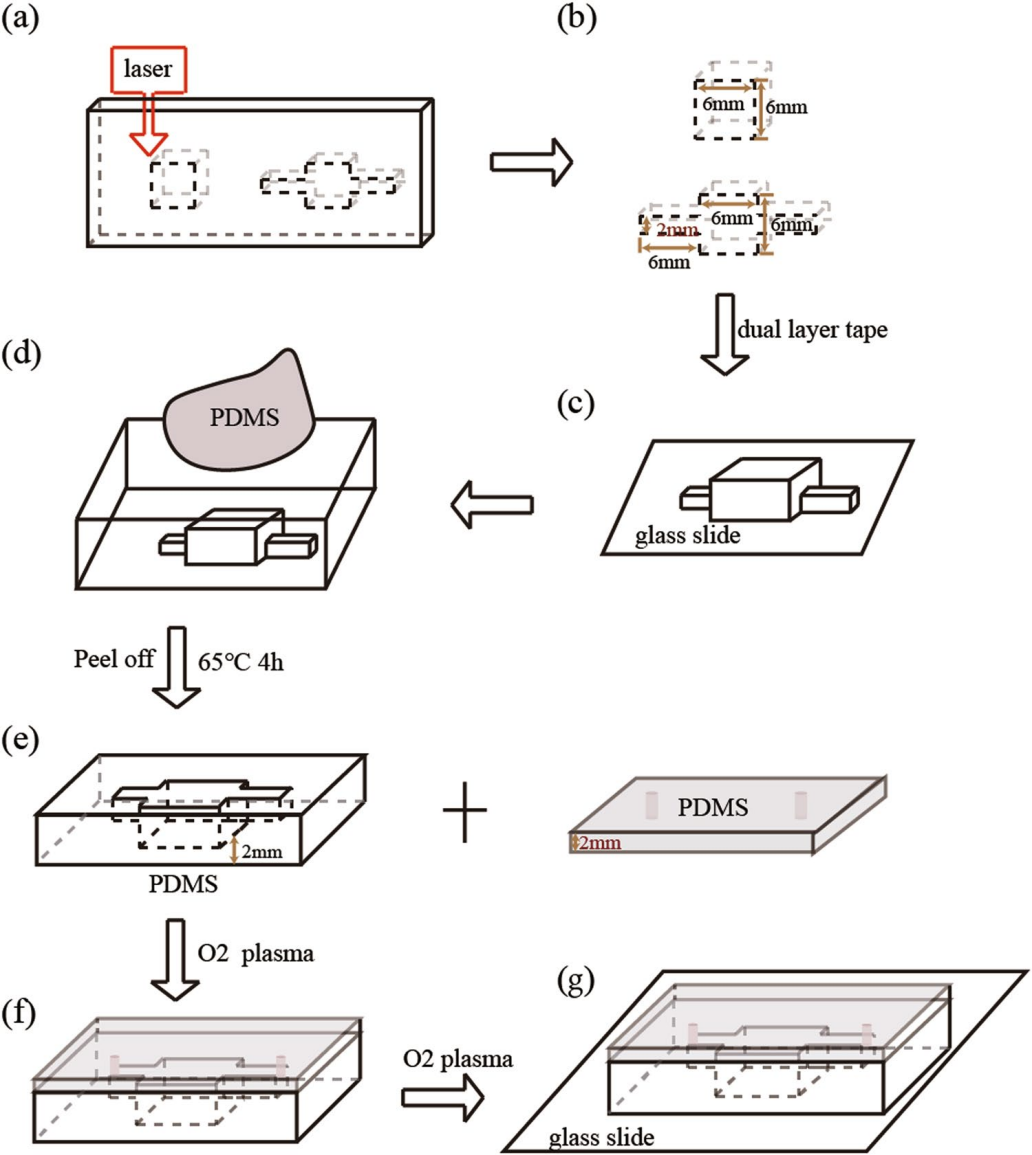
A step-by-step schematic diagram of the production of PDMS devices: (**a**) laser cutting of the PMMA board into the desired trench pattern, (**b**,**c**) fabrication of PMMA mold, (**c**) replication of trench pattern onto PDMS, (**d**) peeling of PDMS chamber from pattern, (**e**) fabrication of plain PDMS with inlet and outlet, (**f**) bonding of two pieces of PDMS by oxygen plasma, and (**g**) bonding of PDMS device to glass slide by oxygen plasma.

The procedures of the fabrication of the trench-shaped PDMS devices are summarized in Fig. 8. Trench-shaped PDMS devices were produced by laser ablation^63^ and imprinting^64^. The building blocks of the poly(methyl methacrylate) (PMMA) mold were designed by a computer-aided design software (AutoCAD 2014, Autodesk, San Rafael, CA, USA) and engraved by a laser ablation machine (Model VLS 2.30, Universal Laser Systems, Scottsdale, AZ, USA) (Fig. 8a,b). The two pieces of PMMA blocks and glass slide were bonded together by double-sided tape, as shown in Fig. 8c. The assembly of PMMA mold with glass slide was settled on a petri dish. PDMS (Sylgard 184, Dow Corning, pre-polymer: curing agent = 10:1) was poured onto the mold, degassed in a vacuum chamber for 30 mins, and cured in a 65 °C oven for 4 hrs (Fig. 8d). After curing, PDMS was carefully peeled off the mold. To prepare an upper seal, a clean glass slide was coated with a 2 mm thick layer of PDMS. Fluid inlets and outlets (1.0 mm OD) were created using a biopsy punch (Miltex, Germany). The two pieces of PDMS were treated with oxygen plasma (PDC-002, Harrick Plasma, high RF power) for 1 min, after which the upper PDMS layer was manually aligned and bonded at 80 °C for 30 mins (Fig. 8f). Then, the PDMS chamber was bonded with a glass slide (Fig. 8g) to enhance resistance against deformation under pressure. Micro Medical Tubing (0.015″I.D. *0.043″O.D.) (Scientific Commodities, Inc., Arizona) was inserted into inlets and outlets. Finally, a thin layer of PDMS was poured on the top layer of the devices to seal the inlet and outlet.

### Isolation of rat primary RGCs and Cell culture

Primary RGCs were isolated from Sprague-Dawley (SD) rats at post-natal day 6 via the magnetic separation method^51,65^. (The animal ethics approval was granted by Animal Experimental Ethics Committee with Ref No. 16-227-CRF, and all experiments were conducted based on the related regulations and guidelines). Firstly, retinal tissues were isolated from the rat eyes. Different retinal cells were dissociated from the tissues by the Neural Tissue Dissociation Kit-Postnatal Neurons (Miltenvi Biotec, Germany) in accordance with the manufacturer’s instructions to obtain a single-cell suspension. RGCs were then isolated from the single cell suspension using Retinal Ganglion Cell Isolation Kit-Rat (Miltenvi Biotec, Germany), according to the manufacturer’s instruction. Briefly, the RGCs were labelled by CD90.1 Microbeads. The negatively selected endothelial cells and microglia were depleted using a magnetic MACSiMAG^TM^ separator. For the positively selected RGCs, the cell suspension was slowly dripped into magnetically bound MS columns (130-04-201, Miltenyi Biotec) to obtain isolated primary RGCs.

The isolated RGCs were plated at approximately 80 cells/mm^2^ within the PDMS devices treated with poly-L-lysine (0.2 mg/mL) and laminin (20 μg/mL) overnight. The culture medium used to culture RGCs was based on the Neurobasal-A medium with supplements, namely, BSA (1%), NaHCO3 (6 mM), B27 (1X), Pen/Strep (1X), GlutaMAX (1X), IGF-1 (10 ng/mL), BDNF (10 ng/mL), CNTF (10 ng/mL) and Forskolin (0.5uM). The medium was replaced every three days. RGCs were cultured in devices at 37 °C with 5% CO_2_ for 3 days before experiment.

The 3D-printed adapter was connected to a plastic column to form the liquid reservoir. The cell culture chambers with RGCs were connected to the liquid reservoir using commercially available syringe needles and medical micro-tubing. After connection, the outlets of each culture chamber were closed by a metallic clamp to avoid outflow of FC-40, which was used to provide the hydrostatic pressure. FC-40 was then infused to the liquid reservoir until the desired liquid level was reached to provide the corresponding hydrostatic pressure for the experiments. The applied hydrostatic pressure is calculated according to Pascal’s law, ${\rm{\Delta }}P={\rm{\rho }}\ast {\rm{g}}\ast ({\rm{\Delta }}h)$ where ΔP is the hydrostatic pressure (Pa), ρ is the density of FC-40 (1.85*10^3^ kg/m^3^) and Δh is the height of liquid above the surface of cell culture solution (m). The corresponding liquid heights of hydrostatic pressure levels 0, 10, 20, 25, 30, 40, and 50 mmHg are 0, 7.4, 14.9, 18.6, 22.3, 29.7, and 37.2 cm, respectively.

### Immunocytochemistry

Primary RGCs inside PDMS chamber were rinsed three times with Hanks’ Balanced Salt Solution (HBSS 1X)(Gibco® by life technologies). RGCs were then fixed with 4% paraformaldehyde solution (PFA) for 30 mins at room temperature. After rinsing, RGCs were incubated with 0.3% Triton in HBSS at room temperature for 15 mins for penetrating the nuclear membrane. The cells were then rinsed with HBSS 3 times again. After that, the cells were blocked with blocking buffer (5% Normal Goat Serum (NGS) + 1% Bovine Serum Albumin (BSA) in HBSS) for another hour at room temperature. The primary Anti-Tubulin beta III isoform Monoclonal antibody (TUJ1) and anti-Brn3a were purchased from Millipore, CA, USA. The primary antibody TUJ1 (1:80) and Brn3a (1:40) were diluted in staining buffer (2% NGS and 1% BSA in PBS) and applied for overnight. Cells were washed with HBSS for 3 times after the removal of the solution with remaining primary antibody. The secondary antibody (anti-mouse green and anti-rabbit red, 1:200) in staining buffer (2% NGS, 1% BSA) was injected into PDMS chamber and incubated for another 2 hours at room temperature with light shielded. After another wash, cells were imaged by a charge-coupled device (CCD) camera connected with an optical microscope (DMIL LED Fluo, Leica Microsystems Inc.).

### Cell image analysis

The whole surface area of primary RGCs inside devices were captured using an optical microscope (Leica Microsystems Inc.). The location of each cell in the device on different days were manually identified. Cell bodies and dendritic branches were manually outlined onto the cell images. For each RGC cell image, the following parameters were measured: (1) total neurites length; (2) the axon length (the longest neurite); (3) cell body area (the area bounded by the cell body contour); (4) branching complexity (measured by Sholl analysis)^50,51,65^. The branching complexity was measured as the number of intersections of the skeletonized neurites for each of the concentric rings with intervals of 6.5 µm, and the first circle was 19.5 µm in diameter. RGCs with a cell body and neurite extensions were counted on day 0 and day 3.

### Statistical analysis

The proportion of RGCs with neurite extensions, the changes of total neurite length, axon length, cell body area, and branching complexity at fixed distances from the cell soma, statistical significance was analysed by one-way ANOVA followed with Bonferroni post-tests between all pressure groups and 0 mmHg, statistical significance was analysed by one-way ANOVA followed with Bonferroni post-tests between all pressure groups and 0 mmHg, 10 mmHg, and 20 mmHg. Linear mixed model was used to calculate the changes of total neurite length, axon length, and branching complexity over time, while controlling for repeated measures per cell. P-value < 0.05 as considered to be statistically significant. All statistical analyses were performed using Prism v.7 (Graphpad Prism, La Jolla, CA, USA) and Stata v.14 (StataCorp, College Station, TX, USA).

### Quantitative analysis on primary RGCs changes under pressure

Primary RGCs were cultured in PDMS devices inside incubator for 3 days for its attachment and neurites growth. Then pressures, namely 0, 10, 20, 25, 30, 40 and 50 mmHg were then applied to cells by the proposed platform. For each pressure condition, we repeated the experiment for at least three times. For each batch of experiments, one to two devices was assigned for the control group (0 mmHg) and one to three devices was assigned for the experimental group. RGCs were isolated independently for each batch of experiment. The whole setup was settled inside an incubator. Cell morphology of primary RGCs were recorded under an optical microscope. The primary RGCs with observable neurites without connections to other cells, were manually selected, followed by outlining of its cell body margin and tracing of all neurite branches. All outlined cell images were loaded a costumed built MATLAB program^50^, the information including the total neurites length, axon length, cell body area and number of intersections of each cell could be obtained.

## Supplementary information

An in vitro pressure model towards studying the response of primary retinal ganglion cells to elevated hydrostatic pressures
