# Supplementary material for: An in vitro pressure model towards studying the response of primary retinal ganglion cells to elevated hydrostatic pressures
Source: Sci Rep. 2019 Jun 21;9:9057. doi: 10.1038/s41598-019-45510-7 (PMC6588599; doi:10.1038/s41598-019-45510-7)
Supplement: Supplementary file 1 — An in vitro pressure model towards studying the response of primary retinal ganglion cells to elevated hydrostatic pressures [file 41598_2019_45510_MOESM1_ESM.docx]

Electronic Supporting Information

**An *in vitro* pressure model towards studying the response of primary retinal ganglion cells to elevated hydrostatic pressures**

Jing Wu,^1,2^ Heather Kayew Mak,^3^ Yau Kei Chan, ^1^ Chen Lin,^3^ Cihang Kong, ^4^ Christopher Kai Shun Leung ^3, *^ and Ho Cheung Shum^1,2,*^

*^1^Department of Mechanical Engineering, University of Hong Kong, Hong Kong*

*^2^HKU-Shenzhen Institute of Research and Innovation (HKU-SIRI), Shenzhen, Guangdong, 518000, China*

*^3^Department of Ophthalmology and Visual Sciences, The Chinese University of Hong Kong, Hong Kong Eye Hospotal 147K Argyle Street Kowloon, Hong Kong*

*^4^Department of Electrical and Electronic Engineering, University of Hong Kong, Hong Kong*


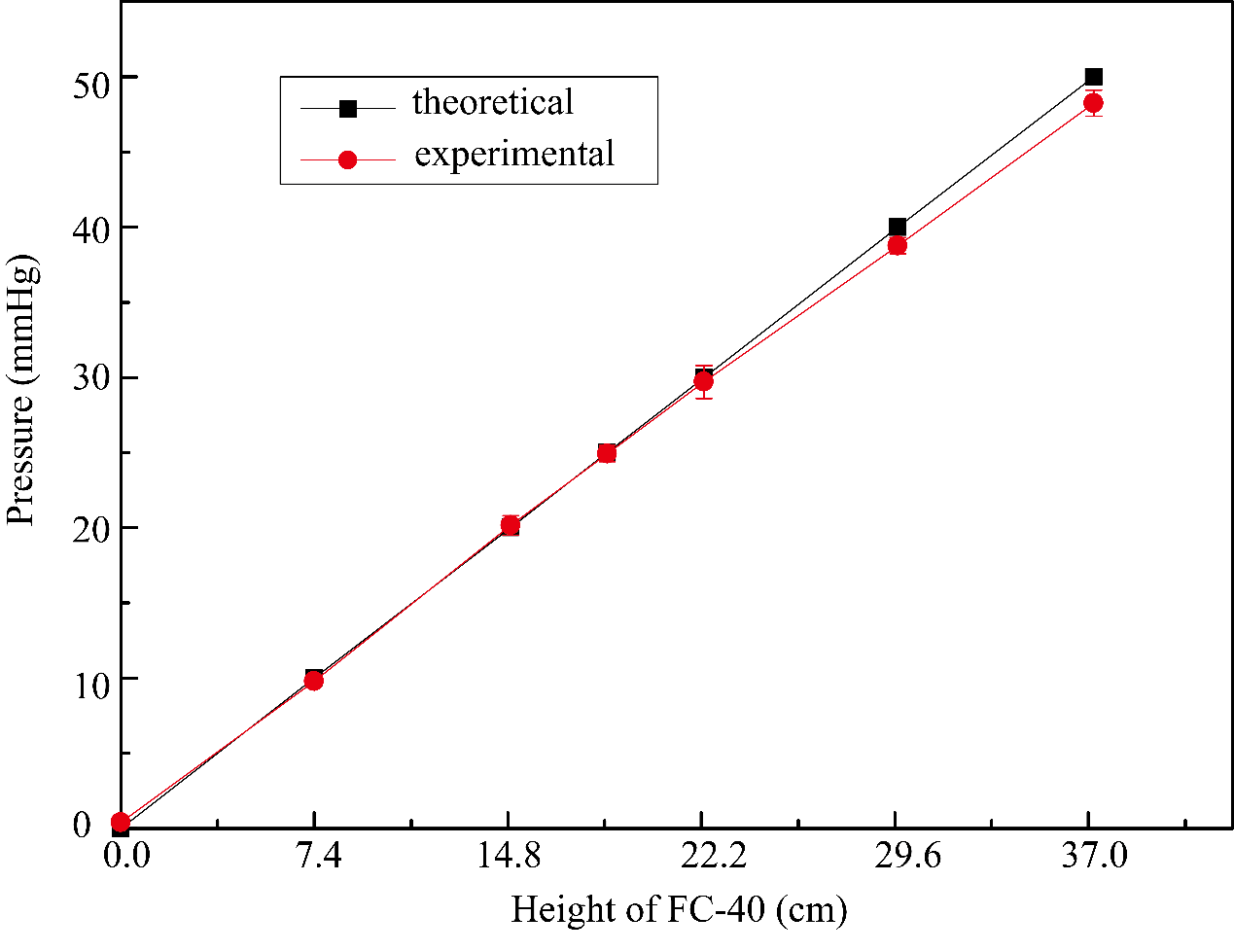


Figure S1, Theoretical and experimental pressure inside PDMS device


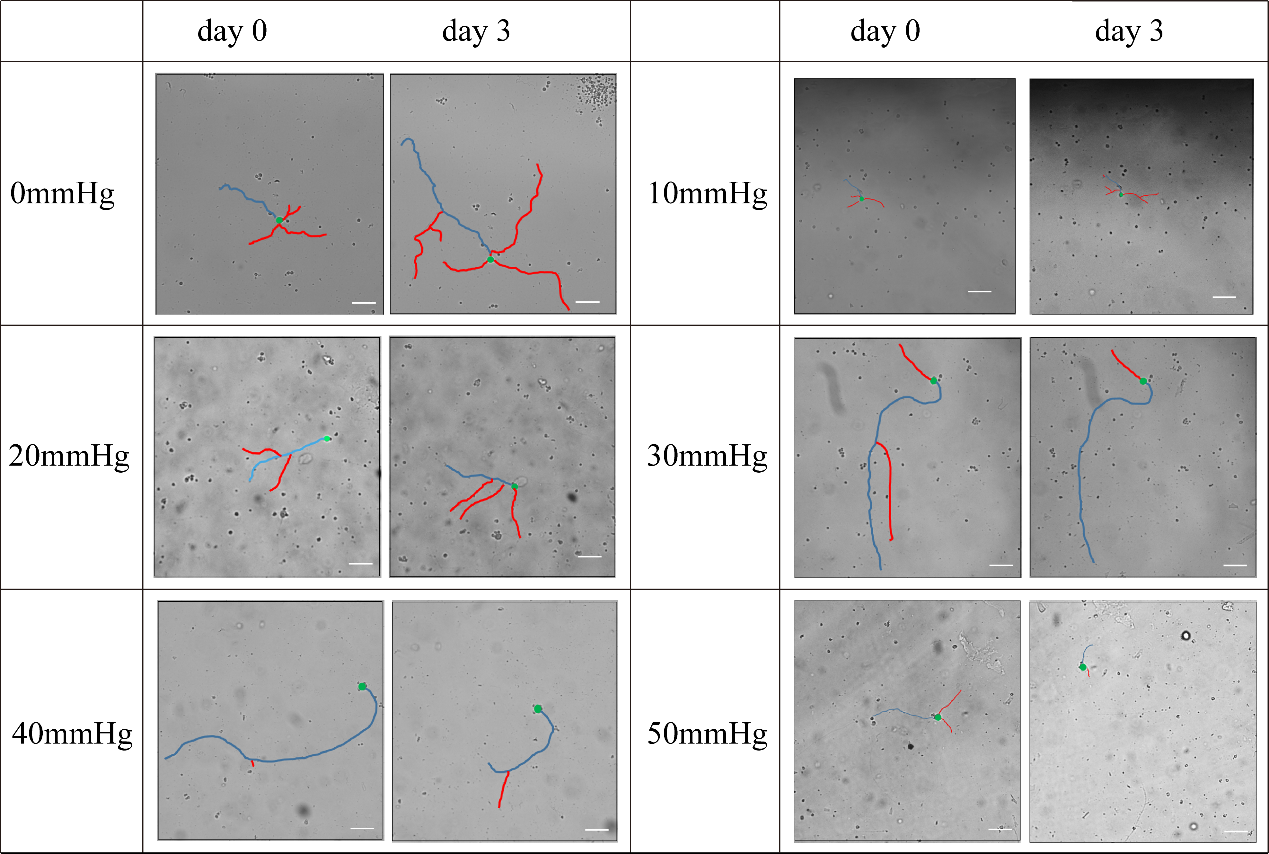


Figure S2 Representative optical images showing primary RGCs 3-days before and after pressure treatment under different pressure levels.
